# Peri-hand space expands beyond reach in the context of walk-and-reach movements

**DOI:** 10.1038/s41598-019-39520-8

**Published:** 2019-02-28

**Authors:** Michael Berger, Peter Neumann, Alexander Gail

**Affiliations:** 1 Cognitive Neuroscience Laboratory, German Primate Center – Leibniz-Institute for Primate Research, Goettingen, Germany; 2 Faculty of Biology and Psychology, University of Goettingen, Goettingen, Germany; 3 Leibniz-ScienceCampus Primate Cognition, Goettingen, Germany; 4 Bernstein Center for Computational Neuroscience, Goettingen, Germany

## Abstract

The brain incorporates sensory information across modalities to be able to interact with our environment. The peripersonal space (PPS), defined by a high level of crossmodal interaction, is centered on the relevant body part, e.g. the hand, but can spatially expand to encompass tools or reach targets during goal-directed behavior. Previous studies considered expansion of the PPS towards goals within immediate or tool-mediated reach, but not the translocation of the body as during walking. Here, we used the crossmodal congruency effect (CCE) to quantify the extension of the PPS and test if PPS can also expand further to include far located walk-and-reach targets accessible only by translocation of the body. We tested for orientation specificity of the hand-centered reference frame, asking if the CCE inverts with inversion of the hand orientation during reach. We show a high CCE with onset of the movement not only towards reach targets but also walk-and-reach targets. When participants must change hand orientation, the CCE decreases, if not vanishes, and does not simply invert. We conclude that the PPS can expand to the action space beyond immediate or tool-mediated reaching distance but is not purely hand-centered with respect to orientation.

## Introduction

Various areas of the primate brain are involved in planning and execution of goal-directed reach movements. Such movements can be directed towards objects within or out of the immediate reach. In the latter case, we also have to plan and execute body movements, e.g. walking, towards this object prior to the reach. It is unclear if the same areas contribute to the spatial cognitive aspects of planning goal-directed walk-and-reach movements to near and far located targets. Patients with brain lesions can suffer from visuo-spatial neglect that is selective for the space either near or far from the body, as shown by a line bisection task executed with a pen or by pointing^1,2^. This suggests that different brain regions are responsible for spatial cognitive encoding of targets near or far from the body. However, when using a tool, a near-space specific neglect can extend into the far space^3^. It suggests that “near” has to be defined operationally, not metrically, and is linked to the space we can interact with. Since full-body movements such as walking increases the space our hand can interact with, the same brain areas that encode immediate reach targets might also encode walk-and-reach targets. Alternatively, this hand interaction space might be constrained to the range of immediate reach and its encoding might be separated from the encoding of walk-and-reach targets. The main goal of the current study is to test behaviorally if such a hand-centered near space can extend towards far located walk-and-reach targets.

The space close to the body with respect to visuo-spatial neglect has been linked to the peripersonal space (PPS)^1–3^. The PPS is defined based on visuo-tactile neurons in premotor cortex^4–6^ and posterior parietal area 7b^5^ which have body-centered visual receptive fields covering the immediate space around the body. The PPS has been proposed to represent an action space in the fronto-parietal circuitry controlling hand and arm movements^7,8^. Planning and execution of visually guided movements are inherently multisensory tasks requiring integration of visual and somatosensory information. Similar to the neglect studies, the spatial extent of the PPS depends on the environment and our ability to interact. It can expand around tools^9,10^, around a video image of the own hand^11^ and around a fake and virtual arm^12,13^.

The PPS has been described with respect to three body-parts: hand (peri-hand space), trunk (peri-trunk space) and head (peri-head space)^8,14–16^. To examine the extent of the PPS in healthy human participants, researchers investigated the effects of multisensory interaction related to the specific body part. Specifically, we are interested in the peri-hand space which can be tested by the crossmodal congruency task^17^. Participants perform a speeded tactile discrimination task, usually to vibro-tactile stimuli at the index finger or thumb (somatosensory component). At the same time, a task irrelevant visual distractor is shown (visual component). Visual and tactile stimuli are spatially congruent or incongruent with respect to the response required by the discrimination task. The crossmodal congruency effect (CCE) is the difference in reaction time, or error rate, in the discrimination task with congruent versus incongruent distractors^18^. With such a task design, the CCE is spatially restricted and strongest for the space immediately around the hand^19^, e.g. when the distractors are placed on the respective fingers next to the vibrators. The spatial range over which the CCE is effective can increase with tool use^20,21^, fake arms^22^, and the mirror-image of the own hand^23^.

More than this, two studies by Brozzoli and colleagues demonstrated that the CCE expands towards a target when participants grasped^24^ or pointed to^25^ a small cylinder with index finger and thumb. While the participants performed the hand movement, they performed the crossmodal congruency task in parallel. Tactile stimulation was either delivered to the moving hand or to the opposite hand as a control. The visual distractor was presented at two different elevations on the target object. At the end of the participant’s movement, the finger was close to the top distractor and the thumb close to the bottom distractor. The visual distractor that was closer to the stimulated finger at the end of the movement was congruent. Crossmodal stimulation was delivered before, at and after the onset of the movement. Brozzoli and colleagues showed that the CCE relative to the moving hand increases already with onset of the reaching movement when the hand has not yet moved closer to the target. This suggests that the peri-hand space can expand towards hand movement targets within reaching distance^26^. However, previous studies did not test if immediate reaching distance marks the boundary for the peri-hand space or if body movements would allow an expansion even beyond the immediate reachable space.

In terms of location (spatial origin of the coordinate system), the hand-related CCE was shown to follow a hand-centered reference frame, since the CCE is strongest when distractors are close to the hand^19^. But the fact that CCE in a reaching context is no longer restricted to the surface of the hand raises the questions in which spatial reference frame “congruency” is defined during reaching. When placing the hand on an object, not just the position but also the orientation of the hand relative to the object needs to be considered. For characterization of this reference frame, orientation and origin of the reference frame matters. Brozzoli and colleagues showed that the hand-centered CCE during goal-directed reaching movements was stronger than before onset of movement when defining congruency in the reference frame of the final hand posture, i.e. when the a congruent distractor light would flash at the respective position at which the finger with the vibrator would land. A strictly hand-centered reference frame would predict that an inversion in hand orientation in its final position inverts this congruency pairing. In other words, if the reference frame of the CCE is defined by the final hand-orientation, then the congruency pairing should be already inverted before and during the movement in cases in which participants have to change their hand-orientation during reach. Alternatively, the congruency pairing is relative to the current hand orientation. In this case, a change in congruency pairing should not be visible before changing the hand posture.

We asked if goal directed walk-and-reach behavior can lead to an expansion of the peri-hand space, measured by the CCE, beyond the immediate reach. In healthy human participants, we measured the CCE before, at and after onset of goal-directed reach or walk-and-reach movements. We report that the CCE increases for walk-and-reach movements already during movement onset just the same as for simple reaches. Furthermore, we tested if the CCE reference frame follows hand rotation. Participants performed the walk-and-reach movement with and without rotating the hand during the movement. We show that this hand rotation leads to a strong decrease, if not elimination, of the CCE.

## Results

**Figure 1:**
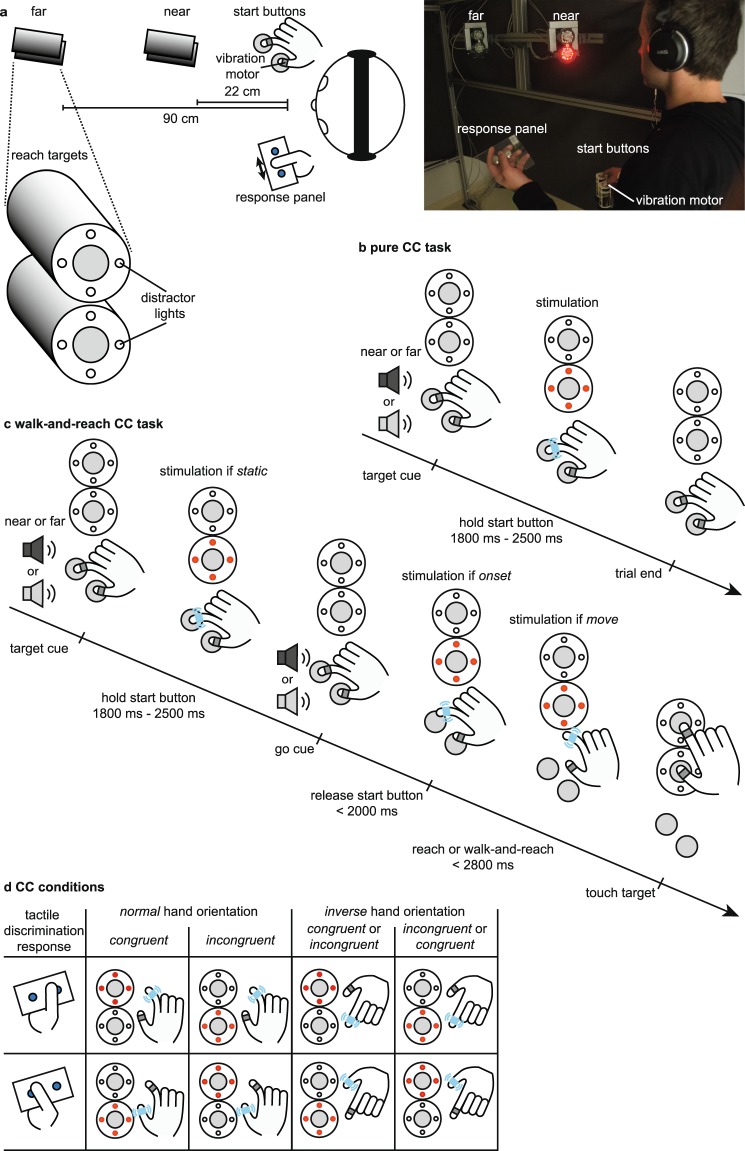
Setup and dual task. (**a**) Setup. The participant stood inside the setup wearing headphones, holding the response panel with two push buttons in the left hand, and touching two touch sensors with right index finger and thumb. Vibration motors were attached to the right index finger and thumb. Near and far targets were in front of the participant each containing two touch sensors and four LEDs around each touch sensor. (**b**) Pure crossmodal congruency (CC) task. The participant had to hold the start buttons and an auditory cue signaled which target is going to be the reach target (*near* or *far*). The participant had to discriminate whether vibro-tactile stimulation was applied to the index finger or thumb by pressing the correct button on the response panel with the thumb of the left hand. A visual distractor was presented on one of the two touch sensors of the cued reach target simultaneously and spatially *congruent* or *incongruent* to the vibro-tactile stimulation. (**c**) Walk-and-reach CC task. The participant had to hold the start buttons and an auditory cue signaled which target is going to be the reach target (*near* or *far*). Upon the second appearance of the auditory cue (go cue), the participant needed to reach to the cued target either with the index finger on the top and the thumb on the bottom sensor (*normal*) or the other way around (*inverse*). The visuo-tactile stimulation could be before (*static*), at (*onset*) or after (*move*) the participant’s hand released the start button. (**d**) CC conditions. The panel displays all possible combinations of *congruent* and *incongruent* visuo-tactile stimulations and the corresponding correct response to the tactile discrimination. The reaching hand could be in a normal or inverted orientation at the target. For inverted hand orientation, which finger-distractor pairing is *congruent* depends on how congruency is defined. We calculated the CCE considering either definition. (Figure drawn by MB, photo by AG).

To investigate visuo-tactile interference during goal-directed reach and walk-and-reach movements, we measured reaction times (RT) and error rates of a tactile discrimination in a crossmodal congruency (CC) paradigm during different phases of a reach or walk-and-reach movement from 59 participants (Fig. 1). Sample size was estimated based on pilot experiments with independent samples. Participants stood within the setup. After an auditory signal had cued one of two movement targets, they waited for the second appearance of the auditory cue (go cue) upon which they performed a reach or walk-and-reach movement to the cued target (walk-and-reach CC task). At the starting and target positions, the participants had to touch two touch sensors with index finger and thumb. For the tactile discrimination performed in parallel, participants had to discriminate between tactile stimuli presented to index finger and thumb of the reaching hand either before (*static*), at (*onset*) or after (*move*) the onset of movement. Simultaneously with the tactile stimulus, participants were shown a visual distractor on the top or bottom part of the selected target. As such, the distractor was either *congruent* or *incongruent* to the stimulated finger. The CCE was quantified as difference in RT between *incongruent* and *congruent* distractor. A subgroup of participants performed reach and walk-and-reach movements either with index finger ending up on the top sensor of the target and thumb on the bottom sensor (*normal*) or the other way around (*inverse*), in different blocks of trials. The *inverse* hand orientation involved a hand rotation during the movement. Since the movement target (*near*/reach or *far*/walk-and-reach) was cued by an auditory signal in the beginning of each trial, participants knew the target and the required hand orientation in the beginning of each trial.

**Figure 2:**
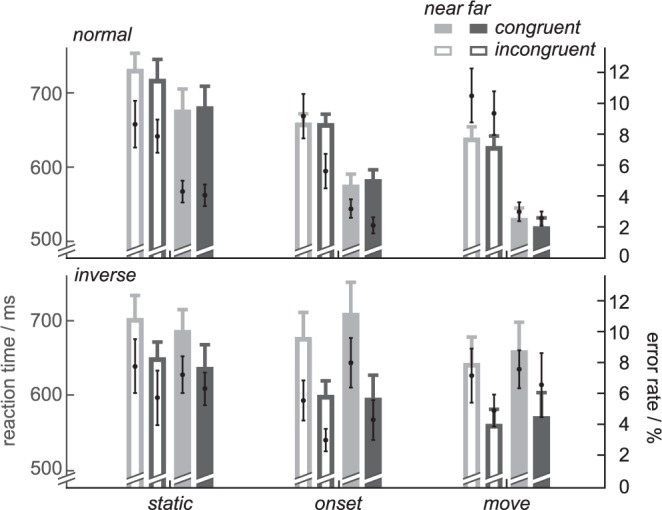
Average reaction times (RT) and error rates for tactile discrimination in the walk-and-reach CC task as function of stimulus timing and hand orientation. The crossmodal congruency effect (CCE) exists for normal hand orientation (top) as *incongruent* (hatched) RT were slower and error rates higher than in *congruent* trials (solid). However, the CCE is not apparent for inverted hand orientation. Bar heights indicate the RT mean, black dots the error rate mean, grey error bars the RT s.e.m and black error bars the error rate s.e.m.

Average RT and error rates for the tactile discrimination in the walk-and-reach CC task are shown in Fig. 2. During normal hand orientation, discrimination in *congruent* trials was reliably faster than in *incongruent* trials for both near and far targets. To quantify our results, first, we calculated a linear mixed effect model (LME) for RT with interacting fixed effects of DISTANCE (*near*, *far*), CONGRUENCY (*congruent*, *incongruent*), TIMING (*static*, *onset*, *move*) and ORIENTATION (*normal*, *inverse*) and a non-interacting fixed effect of experiment PARTICIPATION (“0” first time, “1” second time, etc.; see Methods section). The ANOVA table (Table S1) based on the LME indicated a significant main effect of CONGRUENCY on RT [F(1,702) = 22.97, p < 0.001]. Furthermore, CONGRUENCY significantly interacted with ORIENTATION [F(1,702) = 34.7, p < 0.001] and with ORIENTATION X TIMING [F(2,702) = 3.67, p = 0.026]. This means, as expected, the CCE (main effect of CONGRUENCY on RT) is present, and its strength depends on the task conditions. This is the case for ORIENTATION and TIMING but not DISTANCE as evident by the (lack of) interactions. Similarly for error rates, there was a main effect of CONGRUENCY [F(1,701) = 21.92, p < 0.001] and an interaction with ORIENTATION [F(1,701) = 45.74, p < 0.001] (Table S2). However, CONGRUENCY neither interacted with TIMING nor with ORIENTATION X TIMING. As such, while showing the same trends as RT, error rates in our experiment might not be sensitive enough to uncover all congruency effects. From now on we will focus on RT as a more sensitive measure for our purpose. Detailed ANOVA tables can be found in the supplementary material.

Congruency did not have an effect on the performance of the walk-and-reach movement itself, i.e. start button release time (RTreach) and movement time (MTreach) did not depend on congruency (Fig. S1, Tables S3 and S4).

**Figure 3:**
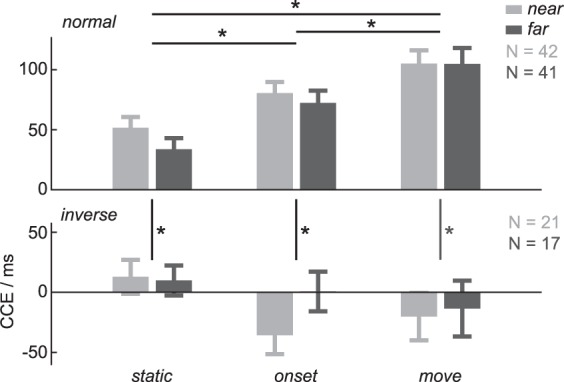
Crossmodal congruency effect (CCE) in the walk-and-reach CC task. No significant differences in the CCE were found between direct reaches (*near)* and walk-and-reaches (*far)*. For normal hand orientation (top) the CCE increased with stimulation at the time of movement onset (*onset*) and during movement execution (*move*), compared to the *static* condition, both, for direct reaches (*near*) and walk-and-reach movements (*far*). The CCE decreased for all conditions when the hand orientation was inverted during movement (here: finger-to-distractor pairing the same as for normal reaches). No significant differences in CCE were found between stimulation times for inverted hand orientation. Bar heights indicate the mean, error bars the s.e.m. Asterisks depict significant differences (p < 0.05) for post-hoc multiple comparisons not including distance (*near*, *far*) as a factor.

To test how crossmodal interaction is influenced by the behavioral task, we computed the CCE, rather than raw RT, for each participant and condition. We calculated an LME to test the dependency of the CCE on DISTANCE, TIMING and ORIENTATION as interacting fixed effects, PARTICIPATION as non-interacting effect, and PARTICIPANTS as random intercept. Average values and their standard errors for each condition are shown in Fig. 3. Violin plots depicting the distribution of the data are shown in the supplementary material (Fig. S2). With this model we addressed two questions. First, does the CCE increase for far located targets with walk-and-reach movements as shown for reach movements to near located targets? In other words, how does the factor DISTANCE influence the data? And second, does the CCE reflect a hand-centered reference frame when the hand orientation changes? In other words, how does the factor ORIENTATION influence the data? We will first present the results for DISTANCE and then for ORIENTATION.

We found no effect of DISTANCE on CCE, neither a main effect [F(1,249) = 0.17, p = 0.68] nor an interaction effect with TIMING [F(2,323) = 1.03, p = 0.36], ORIENTATION [F(1,370) = 2.36, p = 0.13] or both [F(2,323) = 0.67, p = 0.51] (Table S5). This means that there is no indication that the CCE differs between direct reach and walk-and-reach movements.

To test at what time of the walk-and-reach CC task the CCE increased, we performed post-hoc multiple comparison tests based on a reduced model of the CCE without the fixed effects DISTANCE and PARTICIPATION (Table S6). For normal hand orientation, the CCE increased from *static* (LME estimate: 45 ms) to *onset* (LME estimate: 78 ms; difference: 34 ms, p = 0.004) and further from *onset* to *move* (LME estimate: 106 ms; difference: 28 ms, p = 0.024). That means the CCE increases already with onset of the reach and walk-and-reach movement when the hand has not yet approached the near or far located movement target.

Regarding hand orientation, the CCE could be invariant to hand orientation or invert (i.e. rotate) with the hand. To test both cases, we calculated the CCE model twice (Fig. 1d): first, with the previously defined finger-to-distractor congruency pairing leading to a different elevation of the *congruent* finger-distractor pair at the end of the movement for inverted hand orientation; second, with inverted finger-to-distractor pairing (normal Table S5/inverted Table S7). If the CCE of the first model inverts with hand orientation without being affected in strength, then the latter model should show no effect of ORIENTATION. Yet, in both models, there was a significant main effect of ORIENTATION on CCE [F(1,375) = 131.5, p < 0.001/F(1,354) = 125.95, p < 0.001] and an interaction effect for TIMING and ORIENTATION [F(2,323) = 14.59, p < 0.001/F(2,321) = 3.11, p = 0.046]. Thus, inversion of hand orientation does influence CCE but does not simply invert it.

With the post-hoc multiple comparison tests on the reduced model without DISTANCE and PARTICIPATION, there was no significant effect between stimulation times (TIMING) for inverted hand orientation in either model (normal, Table S6/inverted, Table S8). In both models, the CCE decreased for *inverse* compared to *normal* hand orientation: *static* (LME estimate: 9/−16; difference 36/64 ms, p = 0.036/<0.001), *onset* (LME estimate: −22/15 ms, difference: 100/67 ms, p < 0.001) and *move* (LME estimate: −20/12 ms; difference: 126/97 ms, p < 0.001). None of the CCEs with inverted hand orientation are significantly different from zero. This shows that inversion of hand orientation during the movement affects the CCE, but not in a way that would be consistent with an inversion of the hand reference frame.

**Figure 4:**
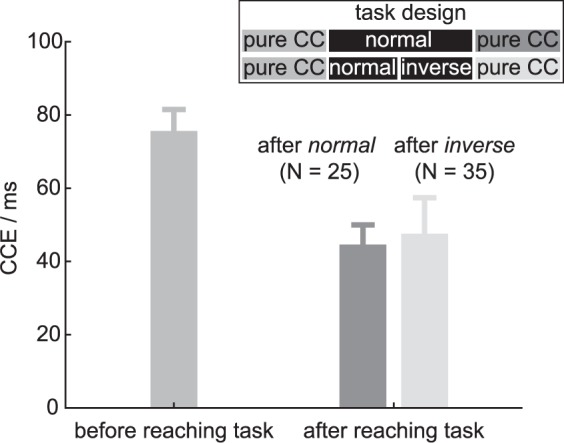
CCE of the pure CC task performed before and after the walk-and-reach CC task. The CCE decreases with practice, but this decrease is not specific to the hand-orientation during practice: There was no significant difference in CCE of a subsequent pure CC task if the preceding block involved *inverse* (light gray) or *normal* (dark gray) walk-and-reach trials. Bar heights indicate the mean, error bars the s.e.m.

The participants performed a pure CC task without reaching at the beginning of the session. At this point, participants had not yet performed any reaches towards the distractor-bearing targets and were not instructed on the walk-and-reach movement either. Still, we could already measure a CCE (Fig. 4). This suggests that a finger-to-distractor association was already established (index finger – top and thumb – bottom). We wanted to know whether the hand orientation, experienced during the walk-and-reach CC task, would affect this prior association. If so, practicing reaching movements with consistently inverted hand orientation should counteract the finger-to-distractor association and decrease the CCE in the pure CC task. To test this, participants performed the pure CC task again at the end of the session after performing the walk-and-reach CC task. One group performed the walk-and-reach only with normal hand orientation, while the other participants performed the walk-and-reach CC task in two blocks, first with normal hand orientation and second with inverted hand orientation. We calculated an LME with the fixed effect ORIENTATION defining whether inverted hand orientation was part of the preceding walk-and-reach CC task of the same session and PARTICIPANT as random intercept. The CCE was smaller in the second pure CC task compared to the first, both, when an *inverse* walk-and-reach block (brightest) and when only a *normal* walk-and-reach block (darkest) was performed in-between. However, there was no significant difference between both orientations [t(82) = 0.03, p = 0.98]. This suggests that reaching practice does not affect the CCE in the non-reaching context in a hand-orientation specific manner.

## Discussion

Previous studies have shown that the interference between tactile information on the hand and task-irrelevant visual information on an object increases from the time of movement onset when reaching with the hand towards the object^24,25^. Our results confirm this change in the crossmodal congruency effect (CCE) for reaching movements. When we compared the CCE in direct reaches to walk-and-reach movements to a target far from the body, we found no difference in CCE between near and far target reaches. To test for the spatial reference frame of congruency in the CCE, participants inverted their hand during the movement so that thumb and index finger touched the target at the location at which for normal hand orientation the respective incongruent distractor light was presented. In the inverted hand condition the CCE decreased, if not vanished, for all timing conditions, but did not invert the congruency pairing between stimulated fingers and distractor light. A decrease in CCE based on practice between before and after the walk-and-reach CC task was independent of the different hand orientations. We conclude that the peripersonal space (PPS), as mapped with the CCE, extends to reach targets beyond the immediate reaching radius during goal-directed walk-and-reach movements.

### Reach vs. walk-and-reach movements

The goal of our study was to test if a visual distractor would affect tactile stimulus discrimination even when being located on a reach target beyond immediate reach, i.e., when participants first have to walk up to the target before reaching it. We could not find any significant differences between reach and walk-and-reach movements in the CCE. As described in previous studies^24,25^, the CCE was already present during movement planning (*static*), and also in the reference condition without any reach movement involved, i.e. in a non-reaching context (pure CC task). The fact that the CCE increased with onset of the movement indicates that the peri-hand space expands with onset of a reaching movement towards the reach target^26^. Based on this interpretation and given that we could not find a difference between reach and walk-and-reach movements, the peri-hand space even extends towards reach goals out of reach and is, as such, independent of a relocation of the body. Note that this extension might not be continuous but an addition to the peri-hand space only around the reach goal. This is suggested by research showing that tool-use leads to an increase in CCE at the tip of a tool but not along the shaft^21^.

Usually the PPS, and correspondingly the CCE, is discussed in context of the reachable space as defined by the space in front of the participant within the immediate reach of the hand^16,26,27^. This emphasis comes from early studies in non-human primates that show two categories of visuo-tactile neurons, one with visual receptive fields close to the skin and the other with visual receptive fields further away but within reaching distance^6,28^. However, in those studies, as well as most other studies in the context of reaching with humans or non-human primates, animals or humans are sitting without having the option to move beyond the reachable space. Body movements towards a target located further away were not considered. Our results suggest that the PPS is defined by the space within we can interact with our environment considering all types of body movements, including walk-and-reach behavior. CCE effects restricted to the reachable space in this sense would reflect the restricted operational space resulting from restrained experimental conditions that do not allow the participant to interact with the space beyond the immediate reach. This view is in line with studies about tool use that show that the PPS can expand beyond the immediate reach around the tool^3,9,10,20,21,29^ and a reach study showing that mirror neurons respond differently when the observed action is blocked by a transparent barrier while still being within arm’s length physical distance^30^. Our behavioral findings add to the converging evidence from multiple lines of research that operational distance rather than physical distance determines the PPS and that this operational distance can be far reaching.

In the present study, we focused on self-initiated movements for which the onset of the body translocation is in parallel with the hand movement onset. It is unclear if the hand-centered PPS expands when the hand is not yet moving but the body already starts moving. For instance when the target is even further away and the hand movement towards the target starts after a couple of steps. Potentially the hand PPS only expands with the movement onset of the relevant effector or with starting any goal directed movement towards the target. It is unclear if the movement needs to be self-initiated, including a strong sense of agency, or can be passively initiated, for instance, when being moved towards a reach target. One study showed that the body centered PPS, measured by auditory-tactile interaction, increased after being passively moved in a wheelchair^31^. This suggests that self-initialization of a movement is not critical for our results. However, whether this holds true for CCE effects related to goal-directed behavior remains to be tested.

### Hand orientation during reaching behavior

The PPS can be centered on different body parts, as suggested by bimodal receptive fields in non-human primates^4,6^, fMRI studies in humans^32,33^, and also spatial properties of behavioral multisensory integration^15,19^. Frames of reference are defined not only by their origin, but also their orientation and scaling of the dimensions. Especially during goal-directed hand movements, the hand orientation needs to match the target object. Since previous CCE studies focused mostly on the distance of the body part from a stimulus or object, conclusions are restricted to the origin of the reference frame. It is not clear from such data how orientation relative to the target object influences the PPS. For example, a hand-centered frame of reference in the strictest sense would predict that the thumb becomes congruent to the top light and the index finger congruent to the bottom when inverting the hand. We show a strong decrease, if not elimination, of the CCE for all stages (*static*, *onset*, *move*) during the walk-and-reach CC task when the participant inverted their hand during the reach (Fig. 3). This means, the CCE was neither invariant to hand orientation, nor did it simply invert with hand orientation. In other words, hand orientation does not affect the CCE in a strictly hand-centered fashion which would be determined by the positions of the fingers on the target after completion of the reach.

A study by Gallace and colleagues investigated the effect of hand orientation in a purely tactile congruency tasks^34^. Similar to the pure CC task, participants performed a speeded vibro-tactile discrimination task on index finger and thumb of one hand. The distractor was a vibro-tactile stimulus on the index finger or thumb of the other hand. Hand orientation of both hands was systematically changed between finger-above-thumb and thumb-above-finger. As shown already by a previous study of the same group, the distractor-stimulus interaction effect was strongest when the stimulus pair had the same elevation independent of orientation of the one or the other hand^35^. This suggests that the tactile representation is encoded in an external reference frame. However when participants performed the same task and reported the stimulated finger verbally instead of using a foot pedal, the stimulus-distractor interaction was highest when the same digits were stimulated, which is a congruency pattern that corresponds to a hand-centered reference frame. In our experiments, participants responded with the left thumb by pressing a button right or left of the thumb, thus, involve a spatial mapping similar to the foot pedal condition. Nonetheless, our results are strongly dependent on hand orientation. Since the reference frame can change dependent on the task, the behavioral task involving hand movements might have induced the hand-centered reference frame. When participants responded verbally, Gallace and colleagues report an asymmetry similar to our results. The stimulus-distractor interaction was lower when the orientation of the two hands was different.

Another study about visuo-tactile interaction found an incomplete inversion of the CCE with hand orientation^36^. Belardinelli and colleagues tested the effect on hand orientation on CCE during the reach to a bottle displayed on a computer screen. Similar to our results, hand orientation affected the CCE before and after movement onset. In contrast to our results, some but not all conditions revealed a significant inverted CCE. One explanation for the stronger hand-orientation effect could be that different visual stimuli (bottle upright and bottle upside-down) were used which could have induced a stronger affordance for the different hand orientation. Still, and like in our results, an inversion of the congruency in the finger-to-distractor pairing following hand rotation cannot fully explain the findings in this previous study.

What could be possible explanations for the asymmetry in the CCE with hand orientation? Staying within the concept of hand-centered reference frames, one could argue that hand inversion was incomplete due to the task design since we did not test different hand orientations at the start of the movement. This is also true for the study of Belardinelli and colleagues. Part of our motivation for this arrangement was to quantify possible dynamics of reference frame changes during reach planning and conduction, which we did not observe. A fully symmetric task design would require that the hand is rotated already at the start instead of performing the hand rotation during the movement. In that case, the CCE might be inverted relative to the normal orientation. If this is true, our lack of an inverted CCE for trials with inverted hand orientation could be a consequence of the start and end position of the hand contributing to the CCE in opposing manner, thereby cancelling each other out.

Alternatively, and independent of the reference frame concept, one could argue that the inverted configuration is less common for this particular target arrangement. The targets were arranged at eye level in front of the subjects. At such elevation above shoulder height, the hands can touch the sensors most comfortably and with the least effort with an “index finger above thumb” orientation. Even before any practice or instruction of the walk-and-reach movement (pure CC task), we measured a positive CCE with distractor lights placed on the vertically oriented near and far targets and with the hand in a horizontal starting position. This suggests that “index finger above thumb” represents a canonical orientation in this setting which is more ergonomic due to the anatomy of the arm and hand. Thereby, this orientation is over-trained during lifetime. Studies with a fake arm model showed that it is possible to embody the fake arm and build a PPS around it in relatively short time and even when the fake arm is detached from the body, but not when it is rotated to be in an unnatural position^12,14,22,37,38^. While embodiment is a result of congruent multisensory stimulation and the present study does not deal with the question of embodiment, the results suggest that multisensory integration might be restricted to plausible body configurations. If the CCE for normal as opposed to inverted hand is an effect of learning/exposure to common postures, it should be possible to also unlearn this association at least partly. Yet, several hundred reaches with inverted hand orientation did not have a significant effect on the end-of-session CCE compared to non-inverted hand orientation. If more practice over multiple days, weeks or months would change this, is unclear. However, the same mechanism that prevents to embody an anatomically unrealistic arm could be responsible for reducing crossmodal interaction for an unnatural reach.

## Conclusion

The CCE increases with goal-directed reaching behavior even before the hand gets closer to the object suggesting a remapping of the PPS to reach goals^24,25,36^. We showed that this holds true even for walk-and-reach movements to reach goals located beyond immediate reach. Furthermore, we tested whether the finger-to-distractor congruency pairing explains CCE effects. When inverting the hand, and thus the finger-to-distractor pairing, during the movement, the CCE decreases but does not simply invert according to the new pairing. We conclude that during planning and execution of goal-directed walk-and-reach movements the PPS expands to the action space independent of whether the movement goal is within immediate reach or not. The hand-related CCE does not only reflect a hand-centered reference frame but changes non-uniformly when the hand is rotated.

## Methods

### Participants

Fifty-nine healthy, right handed (self-reported) participants with normal or corrected-to-normal vision participated in the study (36 f, 23 m; 25 +/− 4 [s.d.] years; 6 have not reported their age). Participants were instructed prior to the experiments and gave their informed consent to take part in this study. Participants received a performance-independent hourly monetary compensation. Experiments were in accordance with institutional guidelines for experiments with humans, adhered to the principles of the Declaration of Helsinki, and were approved by the ethics committee of the Georg-Elias-Mueller-Institute for Psychology, University of Göttingen.

### Experimental setup

Experiments took place in a quiet and dimly lit room. Small vibration motors (3.3 volts DC; Vibration Motor 11.6 × 4.6 × 4.8 mm, Pololu, Las Vegas, Nevada) were taped to the participants’ right index finger and thumb. The strength of the motor was set to its maximum. The vibro-tactile stimulation was above the detection threshold for all participants. We were only interested in reaction time related crossmodal congruency effects and not effects related to detection error. In the left hand, participants held a custom-built response panel with two push buttons (GQ 19H-N, Conrad, Hirschau, Germany) arranged in a way that the left thumb could conveniently rest between and easily touch either button upon request. During the experiment, participants wore headphones (AKG K-182, Harman Deutschland GmbH, Garching, Germany) and listened to Brown noise (generated with “myNoise” and available on https://mynoise.net/NoiseMachines/whiteNoiseGenerator.php). The spectral density of Brown noise is inversely proportional to the squared frequency, i.e. lower frequencies are presented stronger than higher frequencies. Since it is perceived as more pleasant, we chose Brown noise over white noise for which all frequencies are presented equally. The experimenter adjusted the volume of the noise for each participant individually that the participant self-reportedly felt comfortable but could not hear the sound originated from the vibration motors. Auditory cues were also delivered by the headphones.

A total of six (three pairs) capacitive touch sensors (EC3016NPAPL, Carlo Gavazzi, Steinhausen, Switzerland) were mounted at three distances (*start*: 0 cm, *near*: 22 cm, *far*: 90 cm) in front of the participants (Fig. 1a). Touch sensors were offset to the right just far enough that they would not block participants’ straight ahead walking movements. At trial start, participants stood in front of a pair of two touch sensors serving as start “buttons”. The two sensors were mounted at approximately hip level with touch surfaces facing upward at 8 cm horizontal distance center-to-center. They could be comfortably reached with a relaxed right arm and used as resting position for the right index finger and thumb. The other two sensor pairs served as reach targets in front of the participants at two different depths (horizontal start button-to-target distance – *near*: 22 cm; *far*: 90 cm) with vertically oriented touch surfaces facing the participant at 7.5 cm inter-digit separation. Participants could comfortably reach to the near target without moving their shoulder or body. Participants had to make a step forward to conveniently reach the far target. Since we are interested in crossmodal interference beyond the immediate reach, any distance that requires body relocation was sufficient for the *far* condition. Although participants needed to perform only one step, we call this behavior walk-and-reach. The height of the targets was individually adjusted to eye level for each participant. Four synchronized RGB LEDs (WS2812B, Worldsemi Co., Daling Village, China) were located around each touch sensor for delivering the visual distractor cue.

The vibration motors, sensors and LEDs were controlled by a custom-built microcontroller-based interface which in turn was controlled by two custom-written C++ software packages, MoRoCo and MaCaQuE^39^ (MaCaQuE software and schematics available on GitHub: https://github.com/sensorimotorgroupdpz/MaCaQuE), operated on a Mac Mini (Apple Inc., Cupertino, California).

### Experimental task

To measure the crossmodal congruency effect (CCE), participants needed to make a discrimination response on the response panel in their left hand to a 50 ms vibration on their right hand (Fig. 1). More specifically, they were asked to press the button to the left of their left thumb if they felt a vibration on their right thumb and to press the button to the right of their left thumb if they felt a vibration on their right index finger (tactile discrimination). Participants were asked to perform the discrimination task as fast as possible and the first response was registered as long as the next trial did not start yet. At the same time, the participants were instructed by an auditory cue in the beginning of each trial to look at the middle of the *near* or *far* target between the two touch sensors. At the instructed target and simultaneously to the vibration, a task-irrelevant red light was presented for 50 ms either by the LEDs around the upper or the lower touch sensor. The stimuli appeared at least 1200 ms after the instruction which was enough time to focus on the instructed target. Participants were instructed to maintain fixation during the trial.

In the visuo-tactile reference condition (pure CC), participants performed two blocks of trials in a version of the crossmodal congruency (CC) task without movement context (Fig. 1b). Participants conducted the tactile discrimination in combination with visual distractors on the “target” object while holding their right thumb and index finger on the start button, but without executing a reach. The participants were asked to focus on the relevant target which was instructed by an auditory cue in the beginning of each trial. Target (*near*/*far*) and distractor location (top/bottom) were randomly interleaved. One block of the pure CC task was conducted in the beginning, one at the end of each experimental session.

The walk-and-reach CC task, the main part of the experiment, was conducted between the two blocks of the pure CC task (Fig. 1c). It consisted of an instructed-delay reach and walk-and-reach to the cued targets which the participants needed to perform simultaneously with the tactile discrimination. The task contained the following stages after initiating a trial by touching the start buttons: 1) A high or low auditory cue was delivered on the headphones to indicate the near or the far target (DISTANCE); 2) Participants had to keep their hand on the start buttons for another 1800 ms to 2500 ms while focusing on the cued target; 3) When the same sound appeared for the second time (go cue), the participants had to release the start button within a 2000 ms time window and reach to the cued target within a 2800 ms time window; 4) When the participants touched the cued target with both fingers for at least 200 ms, the trial was counted as correct as signaled by a high-pitched tone. If the participants during any stage did not perform the task correctly, the trial was counted as failed which was signaled by a low buzzer tone. Auditory target cues and auditory feedback stimuli on reach performance were all easily distinguishable, as confirmed by oral report of the participants. The participants needed to perform the tactile discrimination in parallel. The vibro-tactile stimulation and visual distractor were delivered once per trial at different time points either during the delay, with onset of the movement or during the movement (see factor TIMING). Participants did not receive any feedback about whether they performed the tactile discrimination correctly or not. Within a block of trials, all conditions ( = combinations of categorical task parameters relevant for this block; see list below) of the combined tactile discrimination and delayed walk-and-reach task were randomized. This was a pseudo-randomization with respect to the performance of the walk-and-reach task: Conditions with a lower success rate appeared more often to obtain an equal number of trials for which the walk-and-reach task was performed correctly. The performance of the tactile discrimination had no influence on the randomization. The duration of the instructed delay was drawn from a homogeneous distribution between minimum and maximum delay (see above), independent of the other parameters. If tactile stimulation was delivered within the instructed delay period, durations before and after the stimulation were drawn from homogeneous distributions between 1200 ms (before)/550 ms (after) and 1550 ms (before)/900 ms (after).

Factor CONGRUENCY: In accordance with the setup of Brozzoli and colleagues^24,25^ and considering that for a reach to our target arrangement the least effortful hand orientation is the index finger on the higher sensor and the thumb on the lower, we defined the congruency of visual distractor and tactile stimulus as follows, *congruent*: upper four LEDs – index finger or lower four LEDs – thumb; *incongruent*: upper four LEDs – thumb or lower four LEDs – index finger. When the hand was inverted during the reaching phase (see below), we tested the CCE under two congruency definitions 1) the same as for normal reaches and 2) with an inverted definition (*congruent*: upper four LEDs – thumb or lower four LEDs – index finger; *incongruent*: upper four LEDs – index finger or lower four LEDs – thumb)

Factor DISTANCE: In the walk-and-reach CC task, at the beginning of each trial, an auditory cue (low or high frequency tone; 235 ms) indicated which target (*near*: reach, or *far*: walk-and-reach) participants had to reach and the distractor would be displayed. If DISTANCE was not randomized in a block, we kept the respective auditory cue to signal the start of the trial. The participants were instructed to keep ocular fixation on a point in the middle between the two touch sensors of the cued target.

Factor ORIENTATION: The walk-and-reach CC task was performed under two different hand orientations, *normal*: touching the top sensor with the index finger and the bottom sensor with the thumb, and *inverse*: touching the top sensor with the thumb and the bottom with the index finger (Fig. 1d). *Normal* and *inverse* reaches were performed in blocks of 120 correct trials ([*congruent*, *incongruent*] x [*static*, *onset*, *move*] x 20 trials) for each of the two conditions ([*near*, *far*]). Thus, participant knew the hand orientation condition from the beginning of each trial.

Factor TIMING: The goal was to measure the CCE at different time points of a goal-directed reach and walk-and-reach movement. The visuo-tactile stimulus pair, to which the participants had to respond as fast as possible, appeared once per trial at three different timing conditions: either 550 ms to 900 ms before the go cue (*static*), at movement onset (*onset*), or during the movement, i.e. 200 ms (*near*) or 450 ms (*far*) after movement onset (*move*). Movement onset was registered online by the release of the start buttons and triggered the *onset* visuo-tactile stimulus with less than 10 ms delay. The time for the stimulation onset in the *move* condition was chosen to be just before target acquisition based on the fastest movement time in a pilot experiment (not shown). For the data presented in this study, this time point corresponded to approximately 24% (*near*) and 38% (*far*) of all movement times and was earlier than the fastest movement time (*near* 266 ms, *far* 481 ms). The inter-trial interval, i.e. the time from trial completion until the new trial could be triggered by the participant, for all tasks was 2 seconds. The next trial started, indicated by the first auditory cue, once the start buttons were touched after the inter-trial interval. A participant’s manual response to the tactile stimulation was accepted as long as it was within the same trial including inter-trial interval, thus, at least up to 2 seconds after the vibration.

In summary, a complete set of all possible experimental conditions comprised in total 480 trials for the walk-and-reach CC task ([*congruent*, *incongruent*] × [*near*, *far*] × [*normal*, *inverse*] × [*static*, *onset*, *move*] × 20 trials) and 160 trials for the two pure CC task (2 × [*congruent*, *incongruent*] × [*near*, *far*] × 20 trials). This number of trials would have been too demanding for the participants to run in a single session. Therefore, we recorded the data in three different experimental sessions. For all sessions, the factors CONGRUENCY and TIMING were fully randomized. The permutations of the factors DISTANCE and ORIENTATION were distributed over separate blocks, i.e. per each block and session one combination of the two factors was used and, hence, the four possible combinations resulted in four separate blocks distributed over two sessions (see Table 1). In the 3^rd^ experimental session the factor DISTANCE was randomized within a block, while ORIENTATION was always *normal*. The sessions and blocks (within a session) are described here in the order in which they were recorded:

**Table 1:** Blocks for each experimental session of the walk-and-reach CC task in original temporal order.

session	block CC before	block walk-and-reach 1	block walk-and-reach 2	block CC after
*Near-normal-inverse:*	pure CC task - near	normal – near	inverse – near	pure CC task - near
*Far-normal-inverse:*	pure CC task - far	normal – far	inverse – far	pure CC task - far
*Near-far-normal (DISTANCE random):*	pure CC task	normal		pure CC task

A pause was introduced between each block of a session. Participants were allowed to practice before each block as long as they needed to feel confident with the task of the upcoming block.

### Exclusion criteria

Each participant’s dataset had to fulfill three criteria to be considered for data analysis:

1. Participants needed to perform at least 20 trials for each condition in which both, the walk-and-reach and the tactile discrimination, were performed correctly. Our aim was to obtain 30 repetitions for each condition. However, we stopped the walk-and-reach blocks earlier for some participants to keep a whole experimental session within 1.5 hours. We chose to include only participants with a minimum of 20 trials per condition but then included all correctly performed trials of these participants in the analysis.
2. Participants should not be biased towards one vibration motor to avoid strategies that favor a motor independent of stimulation condition which might diminish the CCE. To test for such bias, we pooled across all conditions and blocks. As quantification, we defined a modulation index, $$[\mu (R{T}_{indexfinger})-\mu (R{T}_{thumb})]/$$$$[\sigma (R{T}_{indexfinger})+\sigma (R{T}_{thumb})]$$ for which µ is the mean and σ the standard deviation. Participants with a modulation index exceeding the average modulation index across participants by two standard deviations were excluded.
3. For some participants, RT in the *static* condition was unreasonably higher than in all other conditions. We expected that RT could be higher for *static* than for *onset* or *move* due to tactile suppression^40–43^. However, for a few participants, the difference was so high that we suspect participants to have waited purposefully or accidentally for the go cue before responding to the tactile stimulus. As a precaution, we excluded those participants. To identify those participants, we calculated a modulation index again $[\mu (R{T}_{condition1})-\mu (R{T}_{condition2})]/[\sigma (R{T}_{condition1})+\sigma (R{T}_{condition2})]$. We excluded participants with a modulation index exceeding the average modulation index across participants by four standard deviations.

Based on the three criteria, we excluded 4 participants for *near-normal-inverse*, 2 participants for *far-normal-inverse* and 2 participants for *near-far-normal*. Remaining numbers of recorded participants were, *near-normal-inverse*: 21 (15 f, 6 m, 24 +/− 3 [s.d.] years); *far-normal-inverse*: 17 (11 f, 6 m, 25 +/− 3 [s.d.] years); *near-far-normal*: 25 (17 f, 8 m, 26 +/− 4 [s.d.] years). For each session, participants performed all conditions, except of one participant in *near-far-normal* for whom there was a technical failure during the last block (pure CC task). Only few participants performed more than one session: 4 in *near-normal-inverse* and *near-far-normal*; 3 in *near-normal-inverse* and *far-normal-inverse;* 1 in *near-far-normal* and *far-normal-inverse*. For data analysis, we pooled over all sessions and included the information about participant participation in the statistical model (see below).

### Tactile discrimination and reach performance analyses

We measured the reaction time as time between visuo-tactile stimulus and left-hand button press for the tactile discrimination (RT) and the time between go cue and start button release for the walk-and-reach (RTreach). We also measured movement time during the reach (MTreach) as time between start button release and target acquisition. We excluded 9% of all walk-and-reach trials for which the walk-and-reach movement was not performed correctly and 9% of all trials for which no tactile discrimination response was given. We computed each participant’s averages of the three measures separately for each condition. We quantified the CCE based on the difference of the mean reaction times as RT(*incongruent*) – RT(*congruent*).

All data processing was performed using MATLAB (MathWorks Inc., Natick, Massachusetts) and R (R-Foundation, Vienna, Austria). For visualization we used the data visualization toolbox gramm^44^. For statistical analysis, we used linear mixed effect models (LME) using the function “lmer” with maximum log-likelihood estimation from the “lmerTest” library in R. Based on the LME, we generated analyses of variance (ANOVA) tables with Satterthwaite approximation for degrees of freedom using the function “anova” from the “lmerTest” library. Our interpretation if an effect is significant or not is based on those ANOVA tables. For post-hoc multiple comparisons, we perform a generalized linear hypothesis test (function “glht” from the library “multcomp”) on the respective model and extracted adjusted p-values based on a single multivariate test statistic extracted from the model. This method is described in detail in^45^.

We computed LMEs for the observations y of participants’ RT, MTreach, RTreach and CCE, respectively. As fixed effects, we used CONGRUENCY (*congruent*, *incongruent*), ORIENTATION (*normal*, *inverse*), TIMING (*static*, *onset*, *move*) and DISTANCE (*near*, *far*). Since the participants’ performance and the CCE could be influenced by practice^17,46^, we also added PARTICIPATION as fixed effect, which is 0 when participated for the first time, 1 for the second time etc. Participants were used as random intercepts. The model in Wilkinson notation:

$$\begin{array}{c}y\sim ORIENTATION\ast TIMING\ast DISTANCE\ast CONGRUENCY+PARTICIPATION+(1|PARTICIPANT)\end{array}$$

for which y is either RT, RTreach or MTreach. First, we tested whether the CCE was present in our data by testing if CONGRUENCY had an effect on RT. Furthermore, we tested if DISTANCE had an effect on RT. We also tested whether CONGRUENCY had an effect on RTreach or MTreach to see if the CCE influences the reach and walk-and-reach behavior.

After we validated the presence of the CCE, we were mainly interested in effects on the CCE. Accordingly, we computed a LME for CCE:

$$\begin{array}{l}CCE\sim -1+ORIENTATION\ast TIMING\ast DISTANCE+PARTICIPATION\\ \,+(1|PARTICIPANT)\end{array}$$

As there was no effect for DISTANCE and PARTICIPATION, we computed a simplified model without DISTANCE and PARTICIPATION. Finally, we performed post-hoc multiple comparison tests comparing all three stimulation times within normal hand orientation and inverted hand orientation, comparing hand orientations within all three stimulation times and comparing all three stimulation times with inverted hand orientation against the zero-intercept resulting in twelve comparisons. To test for hand orientation specificity of the CCE, we repeated this analysis of the CCE but inverted the congruency pairing of finger to distractor for the trials with inverted hand orientation.

To test whether the training effect on the CCE is different when performing the walk-and-reach CC task with a normal or inverted hand orientation, we tested the effect of the hand orientation in the preceding block on the CCE of the pure CC task after the walk-and-reach CC task. We calculated an LME with ORIENTATION of the preceding block as fixed effect (*normal* for *near-far-normal* and *inverse* for the other two experimental sessions *near-normal-inverse* and *far-normal-inverse*) and PARTICIPANT as random intercept.

## Supplementary information

Supplementary Information

Dataset 1

## Data Availability

All data analyzed during this study are included in the Supplementary Information files.
