# Supplementary material for: Peri-hand space expands beyond reach in the context of walk-and-reach movements
Source: Sci Rep. 2019 Feb 28;9:3013. doi: 10.1038/s41598-019-39520-8 (PMC6395760; doi:10.1038/s41598-019-39520-8)
Supplement: Supplementary file 1 — Supplementary Information [file 41598_2019_39520_MOESM1_ESM.pdf]

Peri-hand space expands beyond reach in the context of walk-and-reach movements

Michael Berger[1,2*], Peter Neumann[1,2], and Alexander Gail[1,2,3,4]

[1] Cognitive Neuroscience Laboratory, German Primate Center – Leibniz-Institute for Primate Research, Goettingen, Germany
[2] Faculty of Biology and Psychology, University of Goettingen, Goettingen, Germany
[3] Leibniz-ScienceCampus Primate Cognition, Goettingen, Germany
[4] Bernstein Center for Computational Neuroscience, Goettingen, Germany

*Corresponding author
Email: mberger@dpz.eu

Supplementary Figures

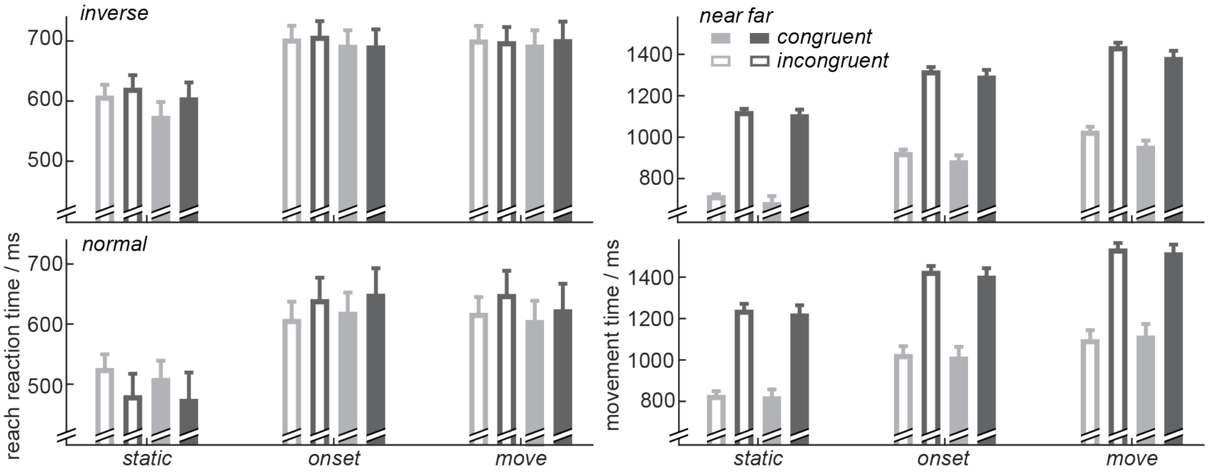

Figure S1: Participants' average reach and walk-and-reach behavior. Left plots show the reaction times in the walk-and-reach task, i.e. the time between go-cue and movement onset (note: RT in the main text refers to the tactile discrimination task). In the *static* condition, the tactile stimulation happened before the go cue. This allowed the participants to fully concentrate on the reaching task afterwards, which might explain the reduced movement reaction times exclusively in this condition. Right plots show the movement times for the walk-and-reach task, i.e. the time between movement onset and target touch. Naturally, walk-and-reach movements (*far*) take longer than just simple reaches. Bar heights indicate the mean, error bars the s.e.m.

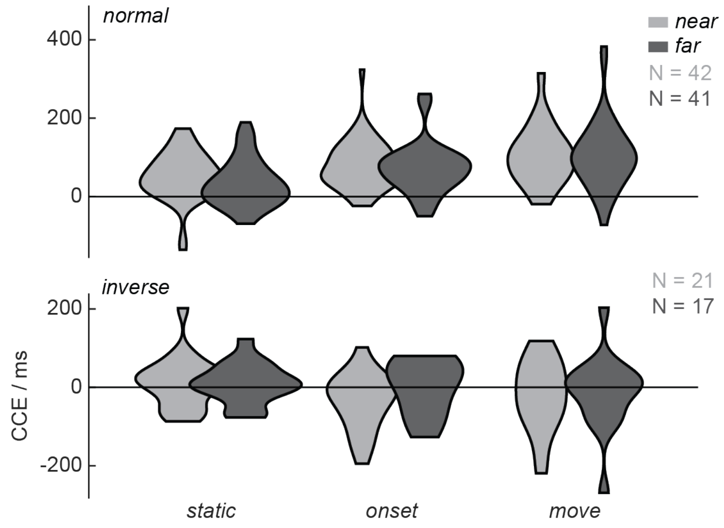

Figure S2: Violin plots showing the distribution of CCEs. It is the same data as presented in figure 3 of the main text.

Dataset

- **Dataset 1** – Mean times (CC task discrimination reaction times ‘RT’, walk-and-reach task reaction times ‘RTreach’ and walk-and-reach task movement times ‘MTreach’) and CC task error rates for each participant and each condition.

Statistic Tables

Analysis of Variance Tables of type III with Satterthwaite approximation for degrees of freedom
Abbreviations of factors:
TIME = TIMING; ORIENT = ORIENTATION; DIST = DISTANCE; CONGR = CONGRUENCY
Significant codes: 0 - '***' - 0.001 - '**' - 0.01 - '*' - 0.05 - '.' - 0.1 - ' ' - 1

Table S1: ANOVA table for reaction time to vibro-tactile stimulation (figure 2)

	Sum Sq	Mean Sq	NumDF	DenDF	F.value	Pr(>F)
**TIME**	896113	448056	2	701.51	58.005	< 0.001 ***
**ORIENT**	138	138	1	726.03	0.018	0.894
**DIST**	11143	11143	1	748.34	1.443	0.23
**CONGR**	177422	177422	1	701.51	22.969	< 0.001 ***
**PARTICIPATION**	361	361	1	755.70	0.047	0.829
**TIME x ORIENT**	128172	64086	2	701.51	8.296	< 0.001 ***
**TIME x DIST**	13465	6732	2	701.51	0.872	0.419
**ORIENT x DIST**	88001	88001	1	738.65	11.393	< 0.001 ***
**TIME x CONGR**	8931	4466	2	701.51	0.578	0.561
**ORIENT x CONGR**	268039	268039	1	701.51	34.700	< 0.001 ***
**DIST x CONGR**	207	207	1	701.51	0.027	0.870
**TIME x ORIENT x DIST**	18392	9196	2	701.51	1.190	0.305
**TIME x ORIENT x CONGR**	56598	28299	2	701.51	3.664	0.026 *
**TIME x DIST x CONGR**	3999	2000	2	701.51	0.259	0.772
**ORIENT x DIST x CONGR**	4870	4870	1	701.51	0.630	0.427
**TIME x ORIENT x DIST x CONGR**	2593	1297	2	701.51	0.168	0.846

Table S2: ANOVA table for error rate of CC task (figure 2)

	Sum Sq	Mean Sq	NumDF	DenDF	F.value	Pr(>F)
**TIME**	0.025775	0.012887	2	700.90	3.748	0.024 *
**ORIENT**	0.000365	0.000365	1	752.77	0.106	0.745
**DIST**	0.006206	0.006206	1	527.91	1.805	0.18
**CONGR**	0.075364	0.075364	1	700.90	21.920	< 0.001 ***
**PARTICIPATION**	0.022617	0.022617	1	596.75	6.578	0.011 *
**TIME x ORIENT**	0.000431	0.000215	2	700.90	0.063	0.939
**TIME x DIST**	0.009354	0.004677	2	700.90	1.360	0.257
**ORIENT x DIST**	0.000331	0.000331	1	755.87	0.096	0.756
**TIME x CONGR**	0.007227	0.003614	2	700.90	1.051	0.35
**ORIENT x CONGR**	0.157247	0.157247	1	700.90	45.737	< 0.001 ***
**DIST x CONGR**	0.002666	0.002666	1	700.90	0.776	0.379
**TIME x ORIENT x DIST**	0.000021	0.000011	2	700.90	0.003	0.997
**TIME x ORIENT x CONGR**	0.011194	0.005597	2	700.90	1.628	0.197
**TIME x DIST x CONGR**	0.000059	0.000030	2	700.90	0.009	0.991
**ORIENT x DIST x CONGR**	0.000677	0.000677	1	700.90	0.197	0.657
**TIME x ORIENT x DIST x CONGR**	0.003819	0.001910	2	700.90	0.555	0.574

Table S3: ANOVA table for reaction time for the start button release (figure S1 left)

	Sum Sq	Mean Sq	NumDF	DenDF	F.value	Pr(>F)
**TIME**	1786371	893185	2	701.46	109.437	< 0.001 ***
**ORIENT**	603865	603865	1	716.94	73.988	< 0.001 ***
**DIST**	17228	17228	1	755.30	2.111	0.147
**CONGR**	5148	5148	1	701.46	0.631	0.427
**PARTICIPATION**	3050	3050	1	751.17	0.374	0.541
**TIME x ORIENT**	37769	18885	2	701.46	2.314	0.1 .
**TIME x DIST**	20796	10398	2	701.46	1.274	0.280
**ORIENT x DIST**	14347	14347	1	727.36	1.758	0.185
**TIME x CONGR**	7256	3628	2	701.46	0.445	0.641
**ORIENT x CONGR**	1980	1980	1	701.46	0.243	0.622
**DIST x CONGR**	379	379	1	701.46	0.046	0.829
**TIME x ORIENT x DIST**	67393	33697	2	701.46	4.129	0.017 *
**TIME x ORIENT x CONGR**	11251	5626	2	701.46	0.689	0.502
**TIME x DIST x CONGR**	2430	1215	2	701.46	0.149	0.862
**ORIENT x DIST x CONGR**	916	916	1	701.46	0.112	0.738
**TIME x ORIENT x DIST x CONGR**	1321	660	2	701.46	0.081	0.922

Table S4: ANOVA table for movement time in the walk-and-reach task (figure S1 right)

	Sum Sq	Mean Sq	NumDF	DenDF	F.value	Pr(>F)
**TIME**	9140317	4570158	2	700.79	438.14	< 0.001 ***
**ORIENT**	1295071	1295071	1	717.74	124.16	< 0.001 ***
**DIST**	8771245	8771245	1	755.89	840.90	< 0.001 ***
**CONGR**	21184	21184	1	700.79	2.03	0.155
**PARTICIPATION**	15445	15445	1	752.83	1.48	0.224
**TIME x ORIENT**	930	465	2	700.79	0.04	0.956
**TIME x DIST**	10489	5245	2	700.79	0.50	0.605
**ORIENT x DIST**	17932	17932	1	728.74	1.72	0.190
**TIME x CONGR**	7313	3656	2	700.79	0.35	0.704
**ORIENT x CONGR**	30069	30069	1	700.79	2.88	0.09 .
**DIST x CONGR**	376	376	1	700.79	0.04	0.849
**TIME x ORIENT x DIST**	335	168	2	700.79	0.02	0.984
**TIME x ORIENT x CONGR**	23418	11709	2	700.79	1.12	0.326
**TIME x DIST x CONGR**	521	260	2	700.79	0.02	0.975
**ORIENT x DIST x CONGR**	10965	10965	1	700.79	1.05	0.306
**TIME x ORIENT x DIST x CONGR**	2988	1494	2	700.79	0.14	0.867

Table S5: ANOVA table for CCE (figure 3/S2)

	Sum Sq	Mean Sq	NumDF	DenDF	F.value	Pr(>F)
**TIME**	17863	8931	3	323.02	2.302	0.102
**DIST**	651	651	1	249.21	0.168	0.682
**ORIENT**	510121	510121	1	375.02	131.505	< 0.001 ***
**PARTICIPATION**	5890	5890	1	269.27	1.518	0.219
**TIME x DIST**	7999	3999	2	323.02	1.031	0.358
**TIME x ORIENT**	113197	56598	2	323.02	14.591	< 0.001 ***
**DIST x ORIENT**	9146	9146	1	370.18	2.358	0.126
**TIME x DIST x ORIENT**	5186	2593	2	323.02	0.668	0.513

Table S6: Post-hoc multiple comparison on CCE ~ -1 + TIME*ORIENT + (1|PARTICIPATION)

	Estimate	Std. Error	z value	Pr(>|z|)
***move/normal – move/inverse* == 0**	126.450	12.631	10.011	< 0.001 ***
***onset/inverse – move/inverse* == 0**	-2.242	14.528	-0.154	1
***static/inverse – move/inverse* == 0**	28.765	14.528	1.980	0.324
***static/normal - move/normal* == 0**	-61.896	9.547	-6.483	< 0.001 ***
***onset/normal - move/normal* == 0**	-28.354	9.547	-2.970	0.029 *
***static/inverse - onset/inverse* == 0**	31.007	14.528	2.134	0.241
***onset/normal - onset/inverse* == 0**	100.338	12631	7.944	< 0.001 ***
***static/normal - onset/normal* == 0**	-33.542	9.547	-3.513	0.005 **
***static/normal - static/inverse* == 0**	35.789	12.631	2.833	0.044 *
***static/inverse* == 0**	8.738	11.320	0.772	0.975
***onset/inverse* == 0**	-22.269	11.320	-1.967	0.331
***move/inverse* == 0**	-20.027	11.320	-1.769	0.456

Table S7: ANOVA table for CCE with inverted CCE definition for inverted hand orientation

	Sum Sq	Mean Sq	NumDF	DenDF	F.value	Pr(>F)
**TIME**	113197	8931	3	320.91	19.725	< 0.001 ***
**DIST**	6305	6305	1	351.50	2.197	0.139
**ORIENT**	361375	361375	1	353.58	125.945	< 0.001 ***
**PARTICIPATION**	180	180	1	367.45	0.063	0.802
**TIME x DIST**	5186	2593	2	320.91	0.904	0.406
**TIME x ORENT**	17863	8931	2	320.91	3.113	0.046 *
**DIST x ORIENT**	908	908	1	360.12	0.317	0.574
**TIME x DIST x ORIENT**	7999	3999	2	320.91	1.394	0.25

Table S8: Post-hoc multiple comparison on CCE ~-1 + TIME*ORIENT + (1|PARTICIPATION) with inverted CCE definition for inverted hand orientation

	Estimate	Std. Error	z value	Pr(>|z|)
***move/normal – move/inverse* == 0**	97.432	10.998	8.859	< 0.001 ***
***onset/inverse – move/inverse* == 0**	2.242	12.504	0.179	1
***static/inverse – move/inverse* == 0**	-28.765	12.504	-2.300	0.176
***static/normal - move/normal* == 0**	-61.896	8.217	-7.533	< 0.001 ***
***onset/normal - move/normal* == 0**	-28.354	8.217	-3.451	0.006 **
***static/inverse - onset/inverse* == 0**	-31.007	12.504	-2.480	0.115
***onset/normal - onset/inverse* == 0**	66.836	10.998	6.077	< 0.001 ***
***static/normal - onset/normal* == 0**	-33.542	8.217	-4.082	< 0.001 ***
***static/normal - static/inverse* == 0**	64.301	10.998	5.847	< 0.001 ***
***static/inverse* == 0**	-16.493	11.079	-1.489	0.665
***onset/inverse* == 0**	14.514	11.079	1.310	0.78
***move/inverse* == 0**	12.273	11.079	1.108	0.884
